# A chicken protein hydrolysate exerts anti‐atherosclerotic effect beyond plasma cholesterol‐lowering activity in Apoe^−/−^ mice

**DOI:** 10.1002/fsn3.1300

**Published:** 2019-12-13

**Authors:** Bodil Bjørndal, Thomas A. Aloysius, Anders Lund, Rasa Slizyte, Pavol Bohov, Ana Karina Carvajal, Rolf K. Berge

**Affiliations:** ^1^ Department of Clinical Science University of Bergen Bergen Norway; ^2^ SINTEF Ocean Trondheim Norway; ^3^ Department of Heart Disease Haukeland University Hospital Bergen Norway

**Keywords:** atherosclerosis, chicken protein hydrolysate, cytokines, fatty acid composition, inflammation

## Abstract

Chicken protein hydrolysates (CPHs) generated from rest raw materials through enzymatic hydrolysis using Corolase PP or Alcalase were shown to reduce inflammation and stimulate hepatic mitochondrial fatty acid oxidation in high‐fat‐fed mice. This study investigates the effect of CPH diets in atherosclerosis‐prone apolipoprotein E‐deficient (Apoe^−/−^) mice. Apoe^−/−^ mice were divided into three groups of 12 animals and fed high‐fat diets with casein (control), Alcalase CPH, or Corolase PP CPH. After 12 weeks, mice were sacrificed, blood samples were collected, and aorta was dissected for subsequent *én face* analysis. Mice fed Corolase PP CPH but not Alcalase CPH had significantly lower % atherosclerotic plaque area in the aortic arch compared to controls (*p* = .015 and *p* = .077, respectively). Plasma and liver cholesterol and triacylglycerol remained constant, but levels of the fatty acid C20:5n‐3 were increased, accompanied by an elevated delta‐5 desaturase index in both CPHs groups. Moreover, a significant reduction of plasma MCP‐1 was detected in Corolase PP CPH compared to control. Overall, our data show that protein hydrolysates from chicken reduced atherosclerosis and attenuated systemic risk factors related to atherosclerotic disorders, not related to changes in the level of plasma cholesterol.

## Graphical abstract

Chicken protein hydrolysates (CPHs) generated from rest raw materials through enzymatic hydrolysis using Corolase PP or Alcalase were shown to reduce aortic atherosclerotic plaque formation compared to a casein control in Apoe^‐/‐^ mice fed a high‐fat diet for 12 weeks. This reduction was independent of plasma lipid levels, but may be linked to effects on fatty acid composition and systemic inflammatory processes.

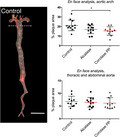

**Abbreviations**
ApoE: apolipoprotein E
CPH: chicken protein hydrolysate
CVD: cardiovascular disease
GM‐CFS: granulocyte‐macrophage colony‐stimulating factor
IFN‐γ: interferon gamma
MCP‐1: monocyte chemotactic protein 1 (CCL2)
NEFA: nonesterified fatty acids
TAG: triacylglycerol
TNF‐α: tumor necrosis factor alpha

## 1 INTRODUCTION

Atherosclerosis is a chronic and progressive disease with a bidirectional interaction between lipids and inflammation as a major feature. The underlying pathological process is lipid accumulation leading to monocyte, macrophage, and T‐cell recruitment, and this slowly progressing chronic disorder of the arteries can ultimately lead to intravascular thrombosis and plaque destabilization with manifestation of acute ischemic events such as myocardial infarction and ischemic stroke. Atherosclerosis‐related cardiovascular disease (CVD) is the leading cause of death in Western countries (Lloyd‐Jones, 2010; Woollard, 2013).

The liver is a central regulator of lipid metabolism and is also reported to be involved in the regulation of the inflammatory state during metabolic disturbances such as fatty liver disease and atherosclerosis (Kleemann & Kooistra, 2005; Rein et al., 2006; Thakur et al., 2012). Indeed, dietary cholesterol absorbed by the liver contributes to inflammation (Kleemann et al., 2007). Additionally, there is growing evidence of mitochondrial dysfunction accompanied by increased oxidative stress in the pathogenesis of atherosclerosis (Lakshmi, Padmaja, Kuppusamy, & Kutala, 2009; Madamanchi & Runge, 2007). The growing epidemic of obesity‐related diseases associated with a metabolic phenotype characterized by dyslipidemia may further contribute to an increased risk of atherosclerosis associated with CVD.

Dietary habits are reported to reduce some risk factors involved in the initiation and progression of atherosclerosis, such as total cholesterol, LDL‐cholesterol, triacylglycerol (TAG), and inflammation. Seafood consumption is considered health beneficial as it lowers plasma lipids and attenuates inflammation (Parolini et al., 2014). This is possibly linked to an increased long‐chain n‐3 PUFAs content of eicosapentaenoic acid (EPA, C20:5n‐3) and docosahexaenoic acid (DHA, C22:6n‐3) and lowering of n‐6 PUFAs such as arachidonic acid (AA; C20:4n‐6). Interestingly, it has also been reported that low levels of circulating eicosatetraenoic acid (C20:4n‐3) and high levels of vaccenic acid (C18:1n‐7) are associated with chronic heart failure (Oie et al., 2011).

It has lately been suggested that some of the advantages of seafood on health could arise from peptides released during digestion, and studies in animals have shown that a number of fish protein hydrolysates have lipid lowering and antioxidant effect (Bjorndal et al., 2013; Wergedahl, Gudbrandsen, Rost, & Berge, 2009; Wergedahl et al., 2004). We have previously reported that a salmon protein hydrolysate has a protective role in atherosclerotic development through mechanisms linked to inhibition of inflammation (Parolini et al., 2014). Recently, we have also found that both the oil and the protein fraction from Antartic krill (*Euphausia superba*) inhibited plaque development in apolipoprotein E (Apoe^−/−^) knockout mice (Parolini et al., 2017). Moreover, we have demonstrated that a fish protein hydrolysate influenced hepatic fatty acid metabolism and fatty acid composition indicating that effects on fatty acid metabolism are important for the bioactivity of protein hydrolysates (Bjorndal et al., 2013; Wergedahl et al., 2009, 2004). Protein from vegetables such as soy, pea, and lupin exerts both TAG and cholesterol‐lowering effects in animals as well as in human studies (Choi et al., 2011; Lee et al., 2009; Morita et al., 1997; Rigamonti et al., 2010). Soy milk and its hydrolysate were also anti‐obesogenic in mice (Choi et al., 2011), and a dietary single‐cell protein produced by bacteria reduced fatty liver in obese Zucker rats (Gudbrandsen et al., 2008). Interestingly, while high‐protein chicken diets have been reported to increase energy intake in rodents (Liisberg et al., 2016), a chicken collagen hydrolysate was shown to exert a strong angiotensin‐converting enzyme (ACE) inhibitory activity and antihypertensive effect as well as to protect from cardiovascular damage in spontaneously hypertensive rats (Saiga et al., 2008; Zhang et al., 2010). Thus, the amount and type of dietary protein and/or hydrolysates may influence systemic inflammation, blood lipid concentrations, and blood pressure and may also have anti‐obesogenic properties.

In this study, we investigated the anti‐atherosclerotic potential of two chicken protein hydrolysates, previously shown to increase mitochondrial fatty acid oxidation and reduce systemic inflammation (Aloysius et al., 2018), on atherosclerotic development in apolipoprotein E knockout (Apoe ^−/−^) mice, characterized by hyperlipidaemia and susceptibility to atherosclerosis (Zhang, Reddick, Piedrahita, & Maeda, 1992).

## 2 MATERIALS AND METHODS

### 2.1 Animals and diets

The animal study was conducted in accordance with the Ethical Guidelines for the Use of Animals in Research (The Norwegian National Research Ethics Committees). The protocol was approved by the Norwegian Food Authorities (Project no. 7618). Female Apoe^−/−^ mice, between 5 and 6 weeks old, were purchased from Taconic (Ry, Denmark). Upon arrival, the mice were placed in open cages, four in each cage, and they were allowed to acclimatize to their surroundings for one week before start of the experiment. The mice were kept in a 12 hr light/dark cycle at a constant temperature (22 ± 2°C) and a relative humidity of 55% (± 5%). During the acclimatization period, the mice had unrestricted access to chow diet and tap water. The cages were block randomized to three different diets, 12 mice per diet. Mice were fed ad libitum, and diet was renewed every 2–3 days. Group 1 (Control) was fed a high‐fat high‐sucrose diet with 41 E% fat, including 0.22% (w/w) cholesterol, and with casein (17 E%) as the protein source (Table 1). In group 2 (Alcalase group), 62.5% of the casein was replaced with CPH generated using the food grade commercial enzyme Alcalase, while in group 3 (Corolase PP group) 62.5% of the casein was replaced with CPH generated using the food grade commercial enzyme Corolase PP. The CPHs were generated as previously published (Aloysius et al., 2018).

**Table 1:** Nutrient composition of the diets in energy % (E%) and weight (g/1000 g)

Nutrients	Casein	Alcalase	Corolase PP
Protein (E%)	17	17	17
Casein (g)	200	75	75
CPH (g)		125	125
Fat (E%)	41	41	41
Soy oil (g)	20	20	20
Lard (g)	190	190	190
Cholesterol (g)	2.2	2.2	2.2
Carbohydrate (E%)	42	42	42
Cornstarch (g)	100	100	100
Dyetrose (g)	50	50	50
Sucrose (g)	340	340	340
Fiber (g)	50	50	50
Micronutrients (E%)	0	0	0
AIN−93G‐MX mineral mix (g)	35	35	35
AIN−93‐VX vitamin mix (g)	10	10	10
L‐Cysteine (g)	3	3	3
Choline bitartrate (g)	2.5	2.5	2.5
Tert‐butyl‐hydroquinone (g)	0.014	0.014	0.014

Note

All ingredients were from Dyets inc. (Bethlehem, PA, USA), except cholesterol and tert‐Butyl hydroquinone, which were purchased from Sigma‐Aldrich (St. Louis, MO, USA).

Abbreviation: CPH, chicken protein hydrolysate.

Mice were fasted for four hours and anaesthetized by inhalation of 2% isoflurane (Schering‐Plough, Kent, UK) before sacrifice. The abdomen was opened in the midline, and EDTA blood was collected by cardiac puncture of the right ventricle and immediately chilled on ice. The samples were centrifuged, and plasma was stored at −80°C prior to analysis. The mice were perfused with 10 ml cold PBS in the left ventricle via 22‐gauge needle and 10‐ml syringe, with an incision in the large liver lobe. Liver and adipose tissues (epididymal, perirenal, and subcutaneous white adipose tissue depots) were collected, weighed, and immediately snap‐frozen in liquid nitrogen and stored at −80°C until further analysis. The aorta was dissected from the heart to 3–5 mm after the iliac bifurcation, and adventitia tissue present was carefully removed. The heart was removed and aortas were fixed for 24 hr in 4% paraformaldehyde in PBS, transferred to PBS, and stored at 4°C.

### 2.2 Én‐face analysis

Aortas were dissected and stained according to Daugherty and Whitman (Daugherty & Whitman, 2003). In short, aortas were placed on a nonreflecting, black surface (a pad of 6 layers of parafilm covered with black vulcanized insulation tape), in a container filled with PBS. The aortas were opened longitudinally under a magnifier (Carl Zeiss OPMI pico) and pinned flat. Aortas were fixed in 70% ethanol for 5 min and stained in 0.25%–0.5% oil red O in isopropyl alcohol for 15 min. Samples were then destained in 80% ethanol for 3 min and rinsed under running water. Stained aortas were stored in PBS until imaging. Images were captured with a digital camera (Canon 5D Mark II). Assessment of % plaque area was performed using the software Image J (http://rsb.info.nih.gov/ij/download.html).

### 2.3 Plasma and liver lipids and fatty acid composition

Liver lipids were extracted from frozen samples according to Bligh and Dyer (Bligh & Dyer, 1959), evaporated under nitrogen, and redissolved in isopropanol before analysis. Lipids from liver were measured enzymatically on a Cobas c 111 system (Roche Diagnostics GmbH) using the triacylglycerol (TRIGL) and cholesterol kit (CHOD‐PAP) from Roche Diagnostics. Plasma lipids were measured enzymatically on a Hitachi 917 system (Roche Diagnostics GmbH) using the triacylglycerol (TRIGL), LDL‐cholesterol (LDLC3), and cholesterol kit (CHOL2) from Roche Diagnostics **(**Diagnostic Systems GmbH), and the nonesterified fatty acid (NEFA) FS and Phospholipids FS kit from DiaSys (Diagnostics Systems GmbH). Total plasma fatty acid composition was analyzed as previously described (Bjorndal et al., 2012).

### 2.4 Plasma inflammatory markers

Plasma concentrations of interleukin 1α (IL‐1α), IL‐1β, IL‐2, granulocyte‐macrophage colony‐stimulating factor (GM‐CSF), interferon gamma (IFN‐γ), IL‐6, monocyte chemotactic protein‐1 (MCP‐1), and tumor necrosis factor‐α (TNF‐α) were determined in animals per group using a custom‐made multiplex MILLIPLEX MAP kit (Millipore Corp) according to manufacturer's protocol. The antibody‐conjugated beads were allowed to react with the sample and a secondary antibody in a 96‐well plate to form a capture sandwich immunoassay. Finally, the concentration of each marker in the solution was determined using a Bio‐Plex 200 instrument, with Bio‐Plex Manager Software 4.1 (Bio‐Rad).

### 2.5 Statistical analysis

Datasets were analyzed using Prism Software 6.0 (Graphpad Software). The results are shown as means of 12 animals per group with their standard deviations, unless otherwise stated. When a one‐way ANOVA test gave a *p*‐value < .05, Dunnett's multiple comparisons test was used to evaluate statistically significant difference between control and treatment groups. *p*‐values < .05 in the post hoc tests were considered statistically significant. Weight change and feed intake over time were evaluated using repeated‐measures two‐way ANOVA (mixed‐model).

## 3 RESULTS

### 3.1 Effects on atherosclerotic development and risk factors related to atherosclerotic disorders

**Figure 1:**
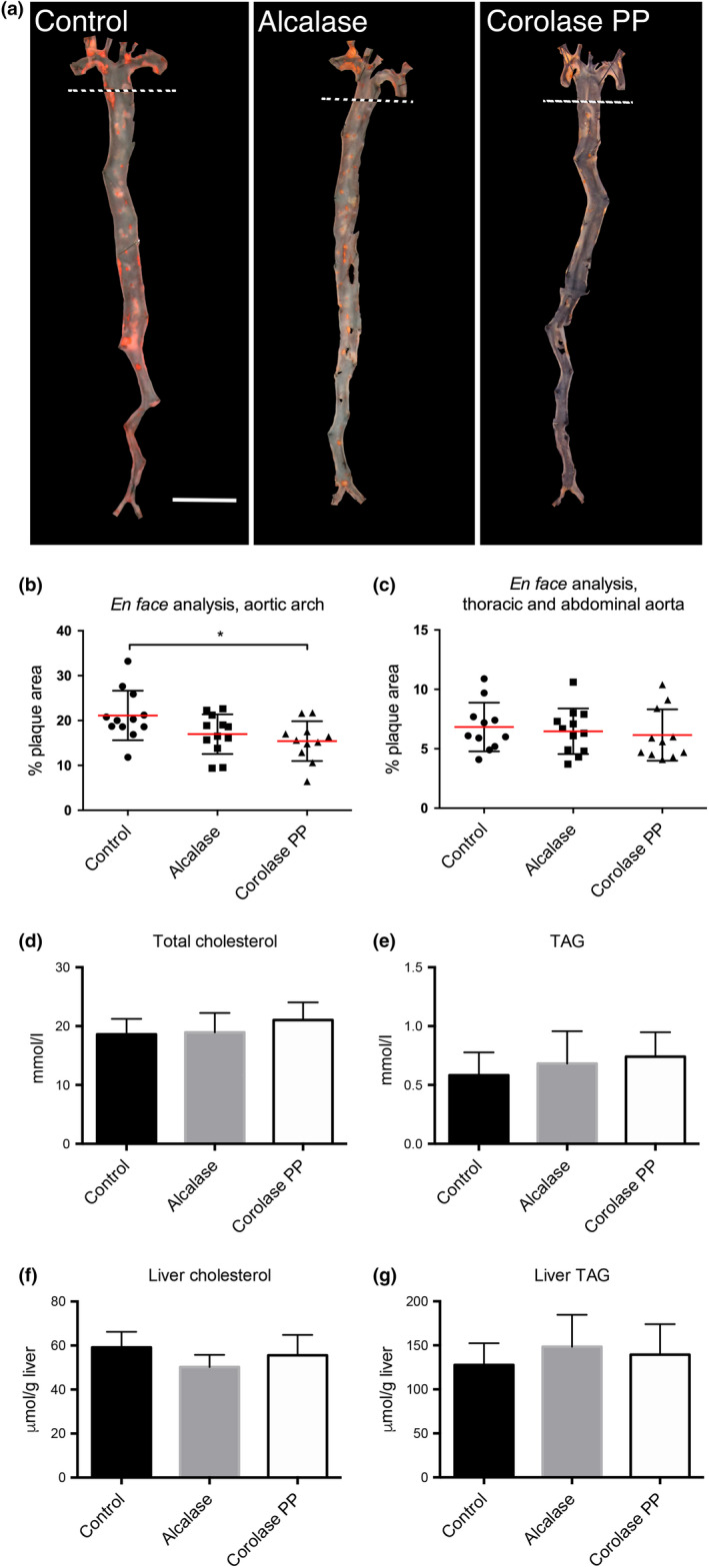
Effect of chicken protein hydrolysates (CPH) produced by Alcalase or Corolase PP on aortic plaque levels and plasma lipids in Apoe^−/−^ mice fed a high‐fat diet for 12 weeks. Representative images of oil red O stained whole aortas are shown (a). Ruler corresponds to 5 mm, and stippled lines indicate the division between arch and thoracic/abdominal aorta. *en‐face* analysis was performed to quantify percent aortic surface area covered by atherosclerotic plaques in the aortic arch (b) or the thoracic and abdominal aorta (c). Levels of plasma total cholesterol level (d), plasma triacylglycerol (TAG, e), liver cholesterol (f), and liver TAG (g) were analyzed. Values are means with standard deviations (*n* = 10–12). Significant difference between groups was determined using one‐way ANOVA with Dunnett's multiple comparisons test (*p < .05)

Enzymatic hydrolysis of chicken rest raw material by Alcalase and Corolase PP generated protein hydrolysates that both affected the hepatic mitochondrial fatty acid oxidation in mice, while Corolase PP CPH had the strongest anti‐inflammatory potential (Aloysius et al., 2018). The data presented here show that after 12 weeks of high‐fat feeding, a significantly lower % plaque area (*p* = .015) was observed in the aortic arch of Corolase PP CPH‐treated Apoe^−/−^ mice compared to control (Figure 1a,b). Conversely, the é*n face* analysis of aortic arch from Alcalase CPH‐fed mice revealed only a trend toward reduced atherosclerosis compared to control (*p* = .077). Low plaque levels were observed in the abdominal and thoracic aorta segments, and no significant difference between the diet groups was observed (Figure 1c). Interestingly, the plasma lipids, including total cholesterol and TAG, were not affected by CPH from Alcalase and Corolase PP (Figure 1d,e). Moreover, no changes in free cholesterol, cholesterol‐ester, NEFA, phospholipids, and LDL‐cholesterol were found between the two protein hydrolysate groups and controls (data not shown). Liver levels of TAG and cholesterol were not significantly different in mice fed the CPH diets compared to the control diet (Figure 1f,g).

**Figure 2:**
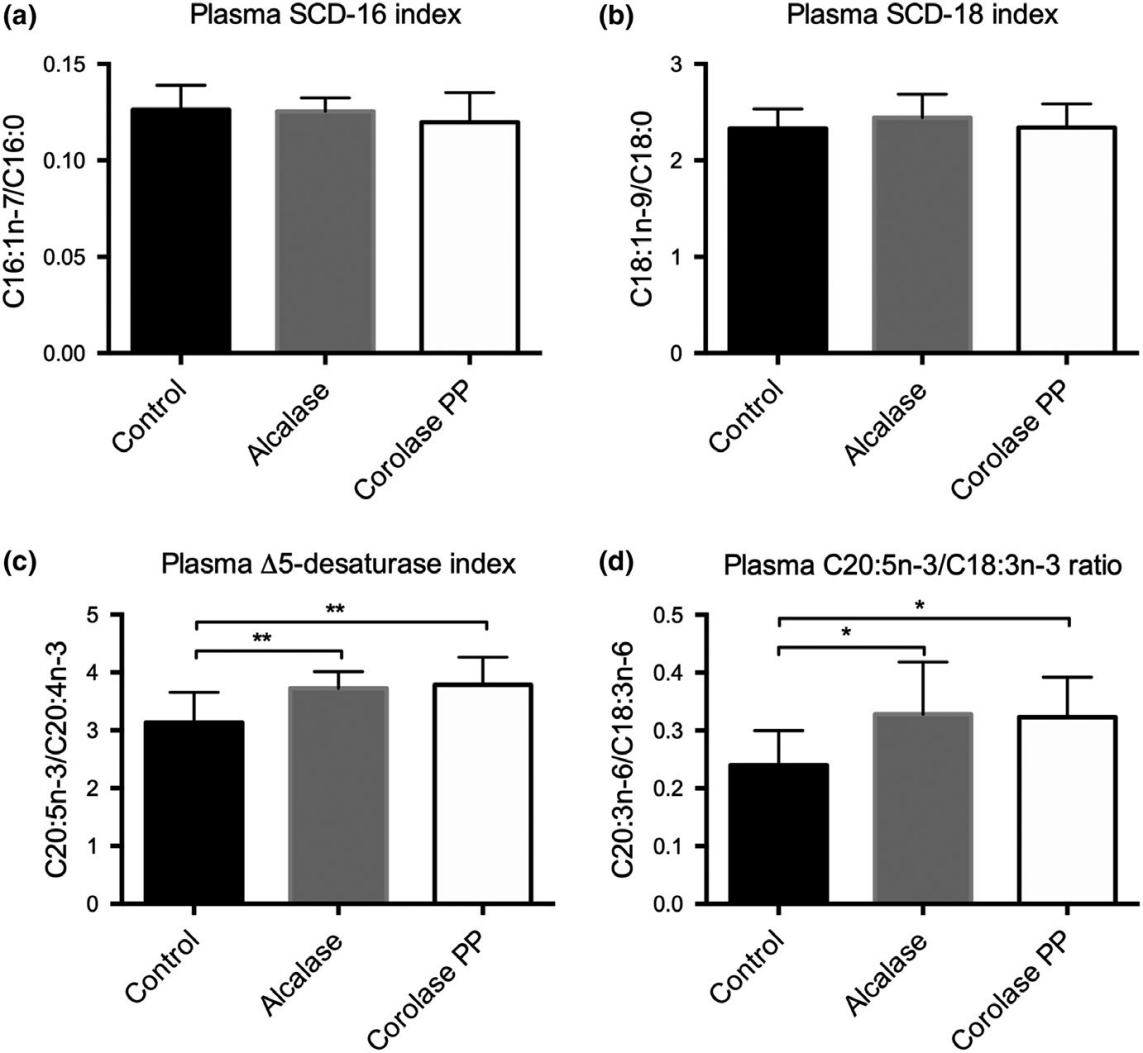
Estimated plasma fatty acid indices in Apoe^−/−^ mice fed a high‐fat diet. (a) The plasma stearoyl‐coenzyme A desaturase 1 (SCD)‐16 index. (b) The plasma SCD‐18 index. (c) The plasma delta 5‐desaturase index. (d) The plasma C20:5n‐3/C18:3n‐3 ratio. Means with standard deviations are shown (*n* = 12). Statistical difference between all diet groups was evaluated using one‐way ANOVA with Dunnett's multiple comparisons test (*p < .05, **p < .01)

The total plasma level of saturated fatty acids (SFAs) and monounsaturated fatty acids (MUFAs) was not changed by the feeding of protein hydrolysates from Alcalase and Corolase PP, but an increase in the levels of long‐chain SFAs and MUFAs was observed in mice fed Alcalase CPH (C20:0, C22:0, C24:0, C20:1n‐9, C20:1n‐7, C22:1n‐9, C22:1n‐7, and C24:1n‐9), and to a lesser degree in mice fed Corolase PP CPH ( C20:0, C22:0, and C22:1n‐9) (Table S1). The estimated activity of stearoyl‐CoA desaturase (SCD)‐16 (C16:1n‐7/C16:0) and SCD‐18 (C18:1n‐9/C18:0) was not changed in either CHP group compared to control (Figure 2a,b). The plasma level of total polyunsaturated (PUFA) n‐3, PUFA n‐6, and PUFA n‐9 was not changed in the two experimental animal groups (Table S1), but the Alcalase CPH reduced the level of total trans fatty acids. Noteworthily, the plasma level of C20:5n‐3 was significantly increased by the Alcalase and Corolase PP hydrolysates (Table S1), followed by a significantly increased delta 5‐desaturase index (C20:5n‐3/C20: 4n‐3) and increased C20:5n‐3/ C18:3n‐3 ratio (Figure 2c,d). Moreover, the plasma level of C18:3n‐6 was significantly increased in the two experimental feeding groups whereas the level of C22:4n‐6 was significantly decreased in the Corolase PP CPH group. The plasma level of C20:4 n‐6 was not changed (Table S1).

**Figure 3:**
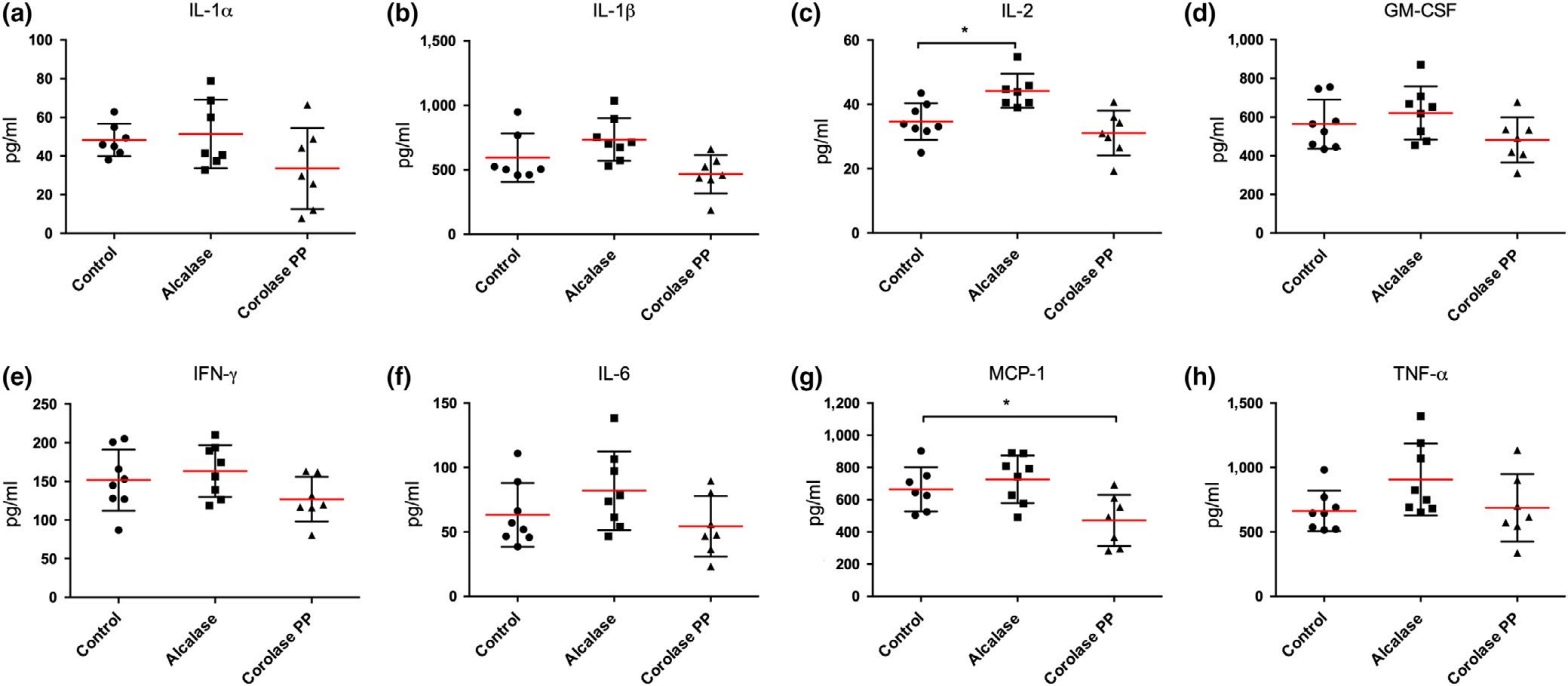
Plasma cytokine levels in Apoe^−/−^ mice fed a casein high‐fat diet (Control) or high‐fat diets with different protein hydrolysates from chicken (Alcalase and Corolase PP). Interleukin IL‐1α (a), IL‐1β (b), IL‐2 (c), granulocyte‐macrophage colony‐stimulating factor (GM‐CSF) (d), interferon gamma (IFN‐γ) (e), IL‐6 (f), monocyte chemotactic protein 1 (MCP‐1/CCL2) (g), and tumor necrosis factor alpha (TNF‐α) (h) were measured by multiplex analysis. Means with standard deviations are shown (*n* = 7–8). Statistical difference between all diet groups was evaluated using one‐way ANOVA with Dunnett's multiple comparisons test (*p < .05)

The protein hydrolysate generated using Corolase PP reduced levels of plasma MCP‐1 compared to control, while Alcalase CPH increased the level of IL‐2 compared to control (Figure 3).

### 3.2 Effects of protein hydrolysates on body weight, feed intake, and adipose tissue mass

**Figure 4:**
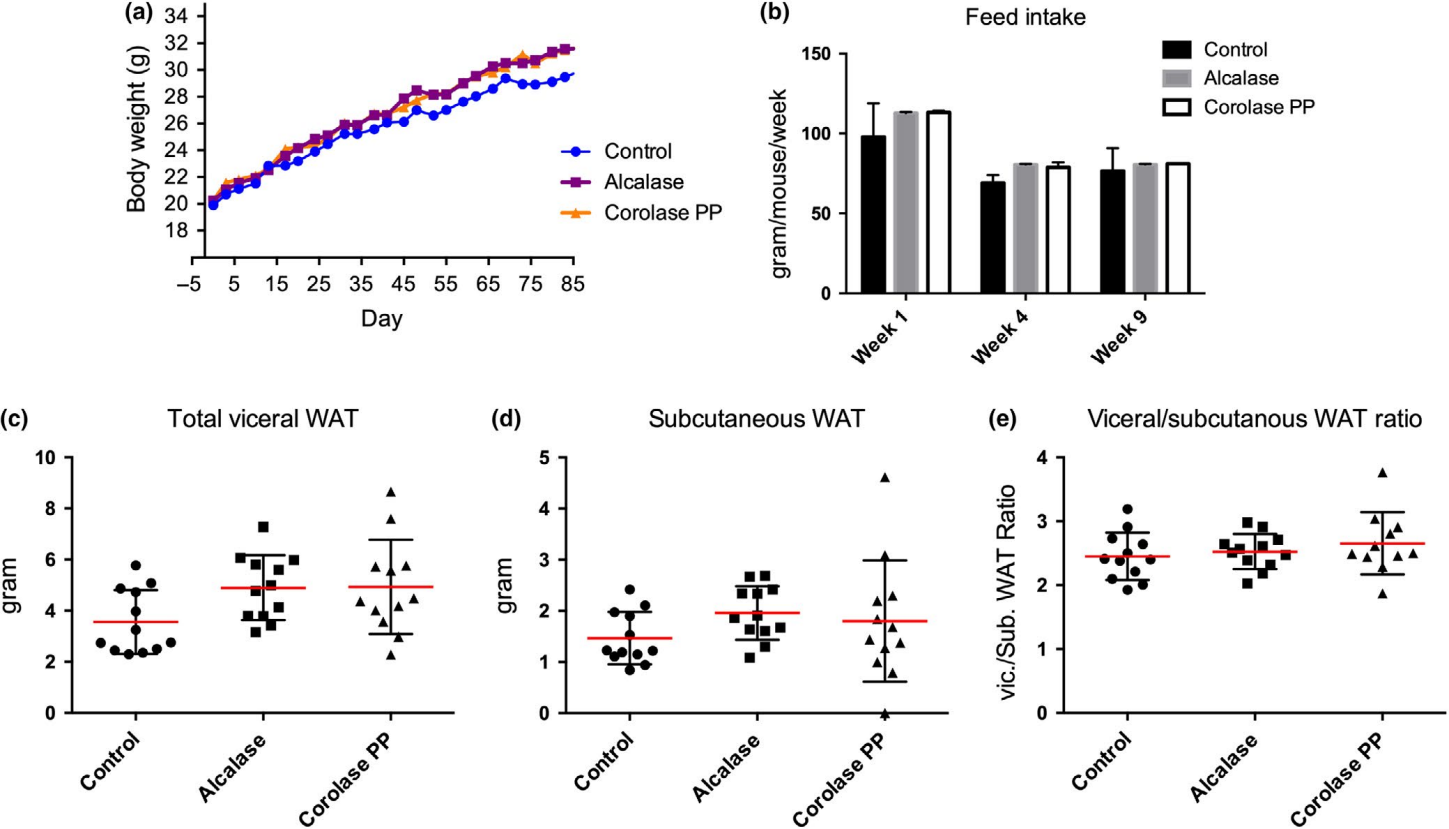
Weight and feed intake in Apoe^−/−^ mice fed a high‐fat diet. (a) Body weight per mouse throughout the study (*n* = 12). (b) Feed intake per mouse during week 1, 4, and 9 of the study (*n* = 3, feed intake average of 4 mice). (c) Weight of total visceral white adipose tissue (WAT; ovarian, perirenal and mesenteric WAT; *n* = 12). (d) Weight of subcutaneous WAT (*n* = 11–12). (e) The ratio between visceral and subcutaneous WAT (*n* = 11–12). Means with standard deviations are shown (*n* = 11–12). Statistical difference between diet groups was evaluated using one‐way ANOVA with Dunnett's multiple comparisons test, while differences in body weight and feed intake in each diet group over time were determined by two‐way repeated‐measures ANOVA (*p* < .05)

The body weight measured at baseline and at different time points during dietary treatment did not show any significant differences among the treatment groups (Figure 4a). The feed intake was measured for one week at three different time points and was not significantly different between the groups (Figure 4b). However, both body weight and feed intake tended to be higher in the two hydrolysate groups compared to control, as did the weights of the visceral WAT depots (*p* = .06) (Figure 4c). Subcutaneous WAT and the ratio between subcutaneous and visceral WAT were not affected (Figure 4d‐e).

## 4 DISCUSSION

Protein hydrolysates may induce health benefits through antioxidative (Raghavan & Kristinsson, 2008), immunomodulating (Zhang et al., 2010), antihypertensive (Saiga et al., 2008), and lipid lowering effects (Erdmann, Cheung, & Schroder, 2008). We have recently shown that a protein hydrolysate from salmon reduced atherosclerosis in Apoe^−/−^ mice, most likely through effects on systemic inflammation (Parolini et al., 2014). Based on previous findings in mice, the amount and type of dietary protein and/or hydrolysates may influence fatty acid oxidation, hypolipidemia, and inflammation (Aloysius et al., 2018; Vik et al., 2015). Therefore, it was of interest to investigate a potential anti‐atherosclerotic effect of protein hydrolysates from chicken previously shown to reduce plasma cytokine levels in a mouse obesity model (Aloysius et al., 2018). Indeed, we here show that Apoe^−/−^ mice fed a high‐fat diet for twelve weeks, containing 12.5% (w/w) protein hydrolysates from chicken generated using the enzyme Corolase PP, developed less atherosclerotic plaques in the aortic arch compared to controls.

Liver is the main organ regulating lipid and lipoprotein metabolism, including plasma TAG and cholesterol levels. It was of interest that the plasma concentrations of cholesterol, TAG and NEFA, and the liver levels of cholesterol and TAG were not affected by CPH treatments in Apoe^−/−^ mice (Figure 1). This was similar to previous findings in a C57BL/6 mice obesity model, where in vitro β‐oxidation was slightly increased but without any effect on hepatic gene levels or plasma lipid levels (Aloysius et al., 2018). However, a changed fatty acid composition was observed in Apoe^−/−^ mice indicating some effect on fatty acid metabolism (Figure 2 and Table S1). After 12 weeks of feeding with protein hydrolysates, the plasma levels of the anti‐inflammatory fatty acids C20:4n‐3 were significantly increased in Corolase PP CPH‐fed mice, while C20:5n‐3 was significantly increased by both the Alcalase CPH‐diet and the Corolase PP CPH‐diet. This was also reflected by an increased delta‐5 desaturase index and an increased ratio of C20:5n‐3/C18:3n‐3 by both CPH diets, indicating an increase in desaturation and elongation processes. Although the plasma level of C18:3n‐6 was reduced by the Alcalase CPH, no significant changes in the major pro‐inflammatory fatty acid, arachidonic acid (C20:4n‐6), were found. Moreover, we have recently reported that low circulating eicosatetraenoic acid (C20:4n‐3) is associated with heart failure (Oie et al., 2011), and this fatty acid was significantly increased by Corolase PP compared to control. The changed plasma fatty acid composition obtained with protein hydrolysates from Alcalase and especially Corolase PP could potentially have been due to increased mitochondrial fatty acid oxidation but was probably not influenced by peroxisomal fatty acid β‐oxidation as previous findings indicated that the acyl‐CoA oxidase 1 (ACOX1) activity is constant in the liver of mice fed these two protein hydrolysates (Aloysius et al., 2018). Supporting this, peroxisomal fatty acid oxidation is involved in chain shortening of long‐chain fatty acid, and we observed an increased plasma level of long‐chain (>C20:0) SFAs and MUFAs in CPH‐fed Apoe^−/−^ mice. Interestingly, Alacalase CPH increased all measured long‐chain SFA and MUFAs compared to control, while the Corolase PP CPH effect was less prominent, which could possibly have influenced the anti‐atherogenic properties of the diets.

Cytokines play a role in the progression of atherosclerosis, and it was of interest that feeding with Corolase PP CPHs, previously shown to reduce plasma cytokine levels in a C57BL/6 mice diet‐induced obesity model (Aloysius et al., 2018), was accompanied by a significantly reduced aortic plaque area, as seen by *én‐face* analysis. The mechanism behind this effect is unclear, but Zhu et al. have shown that marine peptides may act as peroxisome proliferator‐activated receptor ligands (PPAR) and exert anti‐inflammatory effects (Zhu et al., 2010, 2017). In the current study, feeding with Corolase PP CPH only reduced the inflammatory marker MCP‐1, while Alcalase CPH increased the plasma level of IL‐2 (Figure 3). Thus, while this confirmed that Corolase PP CPH had a higher anti‐inflammatory potential than Alcalase CPH, the plasma cytokine lowering was less prominent in high‐fat fed Apoe^−/−^ mice than previously seen in the mouse obesity model (Aloysius et al., 2018). This might have been due to the observed tendency to increased feed intake and increased visceral adipose tissue mass in the CPH‐fed mice, which may have influenced plasma cytokine levels compared to control. It is possible that anti‐inflammatory effects would have been detected in the aortic lesions in the Corolase PP CPH group compared to control and that differences in potency between the Corolase PP CPH‐ and Alcalase CPH‐diet could have been further documented, but unfortunately, we did not have enough material to perform this analysis.

## 5 CONCLUSION

In summary, the present study has some limitation such as absence of data on inflammatory mediators within the aortic lesions, but it gives an indication that peptides generated from chicken rest raw materials may have a protective role in atherosclerotic development through mechanism linked to inhibition of inflammation, not directly related to plasma cholesterol level.

## CONFLICT OF INTEREST

Norilia AS is a partner of the project and has commercial interests in the study product, but was not involved in the study design; in the collection, analysis and interpretation of data; in the writing of the report; or in the decision to submit the article for publication. The authors report no conflict of interest.

## ETHICAL APPROVAL

The animal study was approved by the the Norwegian Food Authorities (Project no. 7,618).
