# Supplementary material for: A chicken protein hydrolysate exerts anti‐atherosclerotic effect beyond plasma cholesterol‐lowering activity in Apoe−/− mice
Source: Food Sci Nutr. 2019 Dec 13;8(7):3052–60. doi: 10.1002/fsn3.1300 (PMC7382182; doi:10.1002/fsn3.1300)
Supplement: Supplementary file 1 [file FSN3-8-3052-s001.docx]

Supporting table 1. Plasma fatty acid composition in in Apoe^-/-^ mice fed a casein high-fat diet (Control) or high-fat diets with different protein hydrolysates from chicken (Alcalase and Corolase PP)^†^.

|  | Control | | Alcalase | | Corolase PP | |
| --- | --- | --- | --- | --- | --- | --- |
| Wt% (g FA/100gTFA) | Mean | SD | Mean | SD | Mean | SD |
| C14:0 | 0.22 | 0.02 | 0.20 | 0.02 | 0.20 | 0.01 |
| C15:0 | 0.12 | 0.01 | 0.11 | 0.01 | 0.11^**^ | 0.01 |
| C16:1n-9 | 0.31 | 0.03 | 0.32 | 0.02 | 0.31 | 0.03 |
| C16:1n-7 | 2.32 | 0.25 | 2.31 | 0.17 | 2.18 | 0.33 |
| C16:1 | 0.014 | 0.002 | 0.013 | 0.001 | 0.013 | 0.001 |
| C16:0 | 18.4 | 0.6 | 18.4 | 0.8 | 18.1 | 0.8 |
| C17:0 | 0.39 | 0.02 | 0.38 | 0.04 | 0.37 | 0.03 |
| C18:3n-6 | 0.13 | 0.01 | 0.15^*^ | 0.02 | 0.15^*^ | 0.02 |
| C18:4n-3 | 0.009 | 0.002 | 0.010 | 0.002 | 0.009 | 0.002 |
| C18:2n-6 | 18.7 | 1.3 | 17.9 | 1.0 | 18.9 | 1.3 |
| C18:3n-3 | 0.83 | 0.10 | 0.76 | 0.09 | 0.80 | 0.08 |
| C18:1n-9 | 28.3 | 0.80 | 28.1 | 1.4 | 27.9 | 1.0 |
| C18:1n-7 | 1.66 | 0.13 | 1.61 | 0.12 | 1.65 | 0.12 |
| C18:1 | 0.15 | 0.01 | 0.13^**^ | 0.01 | 0.14 | 0.01 |
| C18:0 | 12.2 | 0.9 | 11.6 | 1.0 | 12.0 | 1.1 |
| C20:4n-6 | 8.71 | 1.04 | 9.65 | 1.45 | 9.20 | 1.18 |
| C20:5n-3 | 0.20 | 0.04 | 0.24^*^ | 0.04 | 0.25^**^ | 0.05 |
| C20:3n-9 | 0.12 | 0.02 | 0.13 | 0.03 | 0.14 | 0.02 |
| C20:3n-6 | 0.75 | 0.09 | 0.76 | 0.11 | 0.82 | 0.11 |
| C20:4n-3 | 0.062 | 0.005 | 0.065 | 0.009 | 0.067 | 0.006 |
| C20:2n-6 | 0.27 | 0.04 | 0.24 | 0.04 | 0.27 | 0.04 |
| C20:1n-11 | 0.029 | 0.003 | 0.026 | 0.004 | 0.029 | 0.005 |
| C20:1n-9 | 0.25 | 0.03 | 0.29^**^ | 0.04 | 0.28 | 0.03 |
| C20:1n-7 | 0.048 | 0.006 | 0.055^*^ | 0.009 | 0.049 | 0.009 |
| C20:0 | 0.15 | 0.01 | 0.18^***^ | 0.02 | 0.17^*^ | 0.01 |
| C22:5n-6 | 0.12 | 0.04 | 0.10 | 0.02 | 0.09 | 0.01 |
| C22:6n-3 | 2.74 | 0.46 | 3.04 | 0.52 | 2.77 | 0.49 |
| C22:4n-6 | 0.18 | 0.02 | 0.17 | 0.01 | 0.16^*^ | 0.01 |
| C22:5n-3 | 0.15 | 0.01 | 0.15 | 0.02 | 0.15 | 0.01 |
| C22:2n-6 | 0.028 | 0.004 | 0.029 | 0.003 | 0.027 | 0.003 |
| C22:1n-9 | 0.057 | 0.005 | 0.073^***^ | 0.010 | 0.064^*^ | 0.006 |
| C22:1n-7 | 0.022 | 0.004 | 0.028^**^ | 0.004 | 0.023 | 0.004 |
| C22:0 | 0.27 | 0.04 | 0.36^***^ | 0.03 | 0.32^**^ | 0.02 |
| C23:0 | 0.14 | 0.02 | 0.14 | 0.02 | 0.13 | 0.02 |
| C24:1n-9 | 1.04 | 0.15 | 1.37^***^ | 0.13 | 1.15 | 0.16 |
| C24:0 | 0.24 | 0.03 | 0.29^***^ | 0.03 | 0.26 | 0.03 |
| SFA | 32.7 | 0.9 | 32.2 | 1.0 | 32.3 | 0.9 |
| MUFA | 34.2 | 0.9 | 34.3 | 1.4 | 33.8 | 1.1 |
| PUFA n-3 | 3.98 | 0.41 | 4.27 | 0.49 | 4.05 | 0.43 |
| PUFA n-6 | 28.9 | 0.8 | 29.0 | 1.4 | 29.6 | 1.0 |
| PUFA n-9 | 0.12 | 0.02 | 0.133 | 0.032 | 0.14 | 0.02 |
| Trans-FA | 0.10 | 0.01 | 0.09^*^ | 0.01 | 0.10 | 0.01 |
| PUFA n-3/PUFA n-6 | 0.14 | 0.01 | 0.15 | 0.02 | 0.14 | 0.02 |
| ω-3 index | 2.93 | 0.47 | 3.28 | 0.54 | 3.03 | 0.49 |
| D5 desaturase (n-6) | 11.7 | 2.0 | 12.9 | 2.5 | 11.4 | 2.0 |
| D6 desaturase (n-3) | 0.011 | 0.003 | 0.013 | 0.004 | 0.011 | 0.002 |
| D6 desaturase (n-6) | 0.007 | 0.001 | 0.008^*^ | 0.001 | 0.008 | 0.001 |
| C20:4n-6/C18:2n-6 | 0.47 | 0.09 | 0.54 | 0.10 | 0.49 | 0.09 |
| Elong C18:0/C16:0 | 0.67 | 0.06 | 0.63 | 0.07 | 0.67 | 0.08 |
| Elong n-3 C20:4/C18:4 | 7.67 | 2.03 | 6.85 | 1.64 | 7.58 | 1.30 |
| Elong n-6 C20:3/C18:3 | 5.87 | 0.53 | 5.19^*^ | 0.72 | 5.64 | 0.71 |
| Elong n-7 C18:1/C16:1 | 0.72 | 0.08 | 0.70 | 0.09 | 0.77 | 0.12 |
| DBI | 1.32 | 0.05 | 1.36 | 0.07 | 1.35 | 0.05 |

^†^Significant different values compared to control were determined by one-way ANOVA with Dunnett’s multiple comparisons test (*P < 0.05, **P < 0.01, ***P < 0.001).
